# Ethical integration of patient-reported outcomes and digital biomarkers in AI healthcare models: an expert consensus framework

**DOI:** 10.3389/fdgth.2026.1781497

**Published:** 2026-03-10

**Authors:** Joana Seringa, João V. Cordeiro, Rui Santana, Teresa Magalhães

**Affiliations:** NOVA National School of Public Health, Public Health Research Centre, Comprehensive Health Research Center, CHRC, NOVA University Lisbon, Lisbon, Portugal

**Keywords:** artificial intelligence, digital biomarkers, e-Delphi study, ethics, narrative review, patient-generated data, patient-reported outcomes

## Abstract

**Background:**

Alongside expected benefits, several ethical concerns arise from Artificial Intelligence (AI) based models. From the design to the implementation and subsequent evaluation, it is crucial to map potential ethical concerns regarding the use of AI models in healthcare. Patient-Reported Outcomes (PROs) and Digital Biomarkers (DBs) are being increasingly collected to improve patient-centered healthcare systems. However, due to the sensitive nature of this data, its processing into AI models may raise ethical concerns that should be considered. While general AI ethics frameworks exist, no expert consensus has specifically addressed the unique ethical challenges of integrating PROs and DBs in AI healthcare models.

**Objective:**

This study aims to address this gap by establishing expert consensus on ethical, legal, and social considerations for integrating PROs and DBs into AI-driven healthcare models.

**Methods:**

A mixed-method study was performed. Phase 1 consisted of a narrative review to map the ethical landscape and generate an initial pool of recommendations. Phase 2 involved a two-round modified e-Delphi survey to validate and refine these recommendations among a multidisciplinary panel of experts (*n* = 27). The panel included experts in AI, bioethics, clinical research, and data protection, primarily from Southern Europe.

**Results:**

The findings of the two complementary components of this study (narrative review and modified e-Delphi study) were organized around five core ethical principles: autonomy, beneficence, non-maleficence, justice, and transparency and accountability. The modified e-Delphi study achieved high consensus (≥80%) on 55 specific recommendations across these principles. Key recommendations included implementing dynamic consent models, establishing continuous model validation protocols, conducting regular impact assessments, ensuring diverse stakeholder engagement to mitigate biases, and maintaining human oversight within AI systems.

**Conclusion:**

This study provides the first comprehensive expert-validated ethical framework specifically designed for PROs and DBs integration in AI healthcare models, filling a gap in the literature that has primarily focused on general AI ethics rather than the unique challenges posed by patient-generated health data.

## Introduction

Artificial Intelligence (AI) based models promise to improve public health practice and healthcare delivery. AI approaches have been used to predict various health risks and recommend informed decision-making, resulting in more efficient and higher-quality healthcare (1, 2). Nevertheless, adopting AI-based models can potentially elicit significant ethical, legal, and social challenges for individuals, organizations, and societies (3). If neglected or unaddressed, such challenges may result in mistrust or rejection of AI implementation in healthcare and consequent significant opportunity costs. Therefore, a proactive approach toward responsible innovation in this field is needed (4, 5).

Patient-reported outcomes (PROs) measures refer to self-reported instruments designed to measure health, quality of life, health experiences, and related constructs that come directly from patients (6). PROs are increasingly used across health services to improve care (7). As these outcomes focus on measuring the aspects of health that matter to patients, they contribute to more patient-centered healthcare (8). Beyond individual patient care and the benefits of promoting shared decision-making to prioritize, treat, and monitor health status and well-being, collecting PROs may contribute to tracking health outcomes and comparing them to best practices and benchmarks for quality improvement (9, 10). They are also key measures to value-based payment models, population health strategies, health policy, and systems research (11).

PROs can be collected in various environments (e.g., at home, at work, or in the hospital) and methods (e.g., via paper-and-pen, telephone, web-based form, wearable devices, or a mobile app). Therefore, PRO collection infrastructure should be adaptable to meet this diversity (11). In parallel, outcome measures are usually challenging to collect and require adequate resources (12, 13).

Generally, clinical outcomes are recorded in notes, unstructured free text, unstandardized, and variable formats (12). Digital health innovations provide the opportunity to meet this challenge (12) by improving data structure and accessibility and, if correctly designed and implemented, reducing the burden on health professionals and organizations (8). In addition, commercially available electronic devices, such as smartphones, wearables, and Internet of Things (IoT) technologies, allow for capturing and analyzing real-time health-related data, including vital signs, physical activity, stress, or fatigue (12, 14, 15). These outcomes are known as digital biomarkers, whose passive collection depends less on active patient and provider participation. This allows for greater collection consistency and frequency and offers an additional source of patient-centered data (12). Remote and continuous clinical data collection through DBs can enhance diagnostic, monitoring, and therapeutic precision (16, 17) while contributing to empowering individuals to take an active role in managing their health by providing access to personalized health information and facilitating early detection and prevention of disease (15).

Both PROs and DBs collect patient-generated health-related data, which constitutes sensitive personal information, whose processing (from data collection to data storage) should be responsibly designed to guarantee the protection of individuals’ fundamental rights and freedoms (18, 19).

While the ethical implications of AI in healthcare have been comprehensively researched (4, 20–23), the specific challenges posed by integrating patient-generated data, particularly PROs and DBs, in AI models remain underexplored. Unlike general clinical data, PROs and DBs present unique ethical considerations. No previous study has achieved consensus on ethical frameworks specifically tailored to these data types in AI healthcare applications. Therefore, this paper aims to summarize ethical, legal, and social considerations for integrating PROs and DBs in AI-based models for public health and health care and establish consensus on recommendations for their adequate integration.

These considerations are analyzed through the lens of the ethical principles of autonomy, beneficence, non-maleficence, and justice (24). In addition to reviewing the topic through this lens, we also discuss transparency and accountability as relevant principles to AI adoption in healthcare (25).

## Methods

### Study design

**Figure 1:**
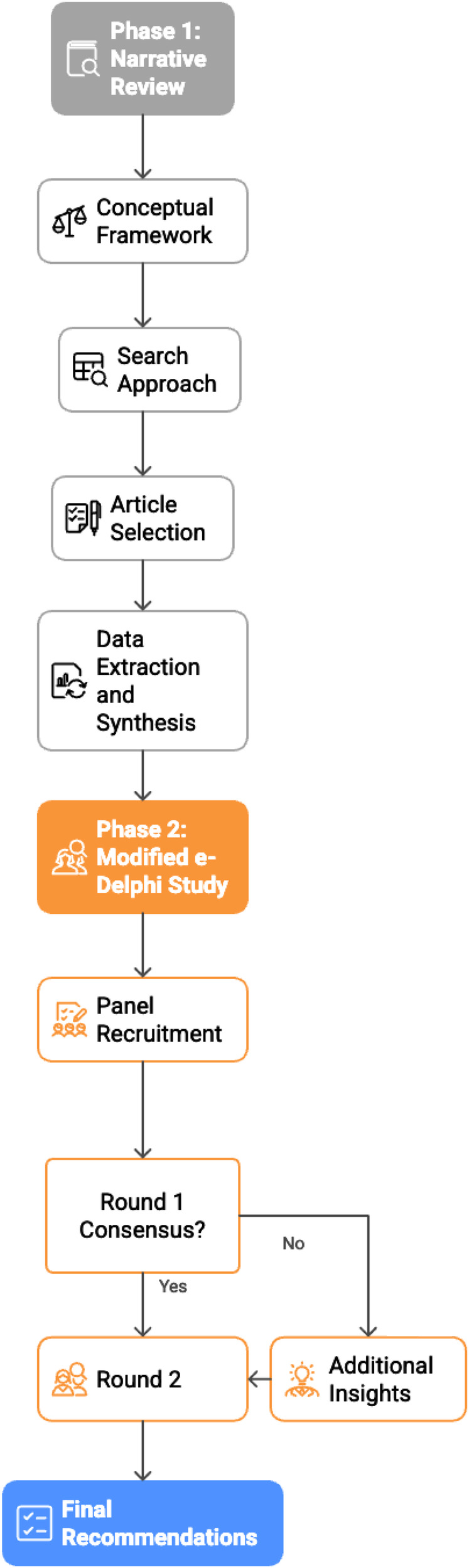
Research methodology flowchart [author's elaboration].

This research employed a mixed-methods approach conducted in two distinct phases. The first phase consisted of a comprehensive narrative review to identify and analyze ethical considerations related to the integration of PROs and DBs in AI-based models in healthcare. Building on these findings, the second phase implemented a modified e-Delphi technique to establish expert consensus on recommendations for ethically sound integration of PROs and DBs in AI-based healthcare models. Figure 1 presents the flowchart of the research methodology.

### Phase 1. Narrative review

#### Conceptual framework

The narrative review was structured around the four foundational bioethical principles established by Beauchamp and Childress (24): autonomy, non-maleficence, beneficence, and justice. This framework was augmented with contemporary ethical considerations of transparency and accountability, which are particularly relevant to AI applications in healthcare (26).

#### Search approach

A targeted literature search was conducted between October 2024 and December 2024 using three electronic databases: PubMed, Scopus, and Web of Science. The search strategy utilized combinations of the following key terms: (1) *Data Types:* “Patient-reported Outcomes”, “Digital Biomarkers”, “Wearables,” “Patient-generated health data”; (2) *Technology:* “Artificial Intelligence”, “Machine Learning”, “Algorithms”; (3) *Ethical Dimensions:* “Ethics”, “Privacy,” “Bias”, “Governance”, “GDPR”, “AI Act”.

No date restrictions were applied. Reference lists of key articles were manually reviewed (snowballing) to identify additional relevant publications.

#### Article selection

Articles were selected based on their relevance to the intersection of AI ethics and patient-generated health data, as determined by the research team. Priority was given to: (a) empirical studies of ethical challenges in AI healthcare implementation; (b) regulatory and policy documents [particularly the General Data Protection Regulation (GDPR) and the European Union Artificial Intelligence Act (EU AI Act)]; (c) theoretical frameworks addressing AI ethics in healthcare; and (d) literature specifically addressing PROs and DBs in digital health contexts.

#### Data extraction and synthesis

Findings were thematically organized according to the five ethical domains (autonomy, beneficence, non-maleficence, justice, transparency/accountability). Key ethical considerations, potential risks, and proposed safeguards identified in the literature were extracted and synthesized to generate preliminary recommendations. This thematic map formed the basis of the statements presented in Round 1 of the Delphi study.

### Phase 2. Modified e-Delphi study

#### Panel recruitment

A purposive sampling approach was used to identify and invite 35 experts with diverse expertise in AI, bioethics, clinical outcomes assessment, digital health, and data protection. Round 1 was completed by 23 participants. To enhance panel diversity and incorporate additional expertise identified during Round 1, four additional participants were recruited for Round 2, bringing the total panel to 27 participants.

The survey was administered through an anonymized platform to encourage candid responses without social desirability bias. To mitigate potential panel discontinuity, invitation reminders emphasized the importance of completing both rounds.

Experts were defined as individuals meeting at least two of the following criteria:

- Minimum 3 years of professional experience in Artificial Intelligence/Machine Learning (AI/ML) applications in healthcare, bioethics, digital health technology development, health data governance, or clinical outcomes research;
- Authorship of peer-reviewed publications, policy documents, or technical reports relevant to AI ethics, patient-reported outcomes, digital biomarkers, or health data governance;
- Professional role involving AI system development, implementation, evaluation, or regulation in healthcare settings;
- Formal credentials in relevant disciplines (e.g., advanced degrees in computer science, bioethics, public health, medicine, health informatics, or law; professional certifications in data protection or health technology assessment).

Patient representatives were invited based on active involvement in patient advocacy organizations or patient advisory councils.

### e-Delphi study

The modified e-Delphi study was conducted between January 2025 and March 2025 and consisted of two sequential rounds:

#### Round 1

- Participants rated their agreement with recommendations derived from the narrative review using a five-point Likert-type scale (1-strongly disagree to 5-strongly agree).
- Open-ended questions allowed experts to provide additional recommendations and contextual insights regarding the processing of PROs and DBs data in AI healthcare models.

#### Round 2

- All items from Round 1, regardless of whether they achieved consensus, were presented again to allow participants to reconsider positions.
- Items were rephrased for clarity where qualitative feedback indicated ambiguity.
- Newly identified recommendations from open-ended responses were incorporated.
- Participants rated agreement using the same five-point Likert-type scale.

### Definition of consensus

Consensus was predefined as ≥80% of participants rating an item as either “agree” (4) or “strongly agree” (5), which aligns with the literature suggesting that an agreement level between 70% to 80% is commonly adopted and regarded as rigorous (27). Items that did not achieve consensus after the second round were noted but not included in the final recommendations.

### Data analysis

Quantitative data were analyzed using descriptive statistics (percentage agreement) for each recommendation. Qualitative data from open-ended responses were analyzed to identify emergent themes and formulate new recommendations for Round 2.

### Ethical considerations and approvals

This study was conducted in accordance with the principles outlined in the Declaration of Helsinki (28). The research protocol received approval from the NOVA National School of Public Health Research Ethics Committee (reference: CEENSP n° 65/2024).

The e-Delphi survey was implemented using a fully anonymous data collection methodology. Participants accessed the survey through a public link that did not require login credentials or email addresses. No personal identifying information was collected at any point during the survey process, ensuring complete anonymity of responses.

All participants provided electronic informed consent prior to participation. The anonymous nature of participation was explicitly stated in the consent information.

All aggregated data from the survey rounds were stored in a password-protected Excel document with access restricted to the research team.

No participant received any type of compensation for taking part in this study.

## Results

### Phase 1: narrative review of ethical, legal, and social considerations of integrating PROs and DBs in AI-based healthcare models

The narrative review identified key ethical themes organized around core ethical principles: autonomy, beneficence, non-maleficence, justice, and transparency and accountability.

#### Principle of autonomy

Respect for autonomy is related to respect and support of autonomous decisions (24). Adopting AI may result in situations where machines are actually or potentially given the responsibility to make important healthcare decisions. According to the autonomy principle, the expansion of AI adoption must respect human autonomy. Furthermore, the notion that AI adoption contributes to patient-centered healthcare suggests that patients should not lose control over their health decisions. In fact, respect for the patient's autonomy consists of involving them in the decision-making process and providing information about the purpose, risks, benefits, and alternatives to an intervention (29).

Therefore, AI systems should assist patients and healthcare professionals in making informed decisions, and human oversight should involve efficient, open monitoring of moral and ethical considerations (21). This is also the case for AI-based models that process PROs and DBs data to provide valuable insights for informed decision-making.

Respect for autonomy also entails safeguarding confidentiality, privacy, and informed consent (21). Patients’ consent for health data processing by AI algorithms must follow a high standard (30). Specifically, obtaining adequate informed consent is imperative for collecting DBs, as the continuous collection of passive data can result in a limitation of autonomy due to unanticipated data treatment (31).

Some authors have already considered that the present informed consent methods for collecting data digitally are unsatisfactory, as patients may not be aware of the type, extent, and potential consequences of the data that is being processed (31). Consenting to collect data digitally is frequently obtained through near incomprehensible terms-of-service agreements, which may lead to consent without previous and precise acknowledgment, which does not meet adequate ethical standards (32).

It is crucial to involve patients through clear and tailored communication and provide technical training to health professionals (33). It should also be considered that informed consent comprehension might be influenced by factors such as age, gender, health literacy, and contextual factors (34).

Recommendations to mitigate these concerns should be in accordance with Article 5 of the GDPR—Principles relating to the processing of personal data (35), including the collection of the smallest and simplest data to meet the purposes of data treatment (“data minimization”); the active engagement of individuals through transparent information regarding collected data, the purposes of the data treatment, and the possible intentional and unintentional impact of data treatment on the individual (“lawfulness, fairness, and transparency”) (18).

Furthermore, dynamic consent models should be adopted whenever possible, including implementing specific options to select data expiration dates (31). In addition, ongoing consent models are recommended to ensure continued comprehension and up-to-date voluntariness (36).

Also, patients must be informed that their data will be gathered for health-related purposes while guaranteeing that data can be treated in an aggregated and anonymous manner for public health purposes, including research (33).

#### Principle of beneficence

AI adoption in healthcare may result in individual and public benefits. The principle of beneficence is related to relieving or preventing harm while balancing benefits against risks and costs (24).

This principle has been associated with the notion of “well-being” in the Montréal Declaration for a responsible development of AI (37), which states that AI systems must promote the well-being of all individuals.

Beyond the individual and public benefit, the beneficence principle also requires strong evidence that the benefits will exceed the risks (38) in individuals and communities (30).

The risk-benefit ratio assessment evaluates the benefits of AI adoption and its potential harms. This assessment should be based on a human-centered design approach, whereupon the people for whom the algorithm (or other digital solution) is being developed are involved and guide the design process from the beginning and to whom the final solution responds (30).

Beyond the initial assessment of the risk-benefit ratio, the AI algorithm's continuous learning characteristic requires continuous evaluation of whether AI adoption results in patient benefit (39) and whether such benefit outweighs the risks.

Other possible benefits of integrating PROs and DBs in AI-based models include providing personalized care and anticipating healthcare needs (individual benefit), cost reduction, and increased quality performance (public benefit) (40).

In summary, the burden of collecting both PROs and DBs must be weighed against the benefits of collecting them.

For AI adoption in healthcare to yield individual and public benefits, it is crucial to ensure seamless interoperability and effective data sharing across systems. Interoperability, the ability of different healthcare systems and technologies to communicate and exchange data, is essential for the efficient deployment and integration of AI solutions (41).

In line with the principle of beneficence, interoperability and secure data sharing facilitates better communication between healthcare providers, patients, and AI systems, allowing for a more coordinated approach to care that maximizes benefits and minimizes risks (42).

#### Principle of non-maleficence

The principle of nonmaleficence is related to avoiding the causation of harm (24).

Healthcare data are some of the most sensitive information about a person. Respecting a person's privacy is a fundamental ethical principle because it is directly linked with patient autonomy, personal identity, and well-being (43).

PROs and DBs, as healthcare-sensitive information, raise ethical, legal and social concerns regarding their collection, storage, access, and sharing (18).

Specifically, DBs data is collected and shared from personal devices, whose encryption methods may not be sufficient to guarantee data privacy (44). Therefore, data must be stored securely, preventing unauthorized access and possible misuse (also considering respect for the principle of non-maleficence). Several regulations (35, 37, 45) emphasize implementing procedures to guarantee data confidentiality. Furthermore, data controllers should carefully determine access levels granted to other stakeholders while considering and respecting autonomy, the right to privacy, and legitimate uses of data (33). Also, individuals must maintain control over their personal data, especially regarding its collection, use, and dissemination (35, 37).

These and other concerns led to important initiatives such as the Global Pulse Data Privacy and Data Protection Principles from the United Nations and the General Data Protection Regulation (GDPR) from the European Union, both adopted in 2018 (30). The GDPR establishes a legal foundation for data protection by encouraging a more proactive, systematic, and comprehensive approach (31). This regulation thus expresses a legislative initiative to address emerging digital challenges regarding personal data (46). The European Union (EU) Data Act, which entered into force on January 11, 2024, and complements the Data Governance Act (47), is also a significant development in the European Union's data landscape that aims to ensure fair access to and use of data across all economic sectors, including healthcare, while ensuring protection of personal data (45).

The Data Act aligns with the European Health Data Space (EHDS), providing secure access to a wide range of health data. Its main goal is to empower individuals to take ownership of their health data while allowing the EU to fully utilize the potential provided by a safe and secure exchange, use, and reuse of health data (48).

In addition, the AI Act, proposed by the European Commission in 2021 and unanimously endorsed by the EU 27 member states in December 2023 and recently entered into force, is the first comprehensive AI law to regulate AI use in the European Union (49). This legal document addresses several ethical, legal and social implications of AI, including those related to adopting AI-based models (49). The AI Act classifies AI systems based on a ‘risk-based approach’ and mandates various development and use requirements in multiple sectors, including healthcare (50). It emphasizes the need for transparency in AI systems and addresses privacy and security concerns (50), which would be relevant to data preprocessing.

Even with ongoing improvements in AI and data accessibility, AI-based solutions in healthcare may be linked to malfunctions and errors that raise questions about patient safety, including (1) false negatives, such as missed diagnoses of life-threatening conditions; (2) false positives, leading to unnecessary treatments; and (3) inappropriate interventions, for example, caused by inaccurate diagnosis or incorrect prioritization of interventions (51). These situations might be related to differences between data used to train an algorithm and real-world data encountered during clinical deployment, highlighting the need for broad testing and validating AI algorithms in diverse patient populations (29).

When collecting DBs for AI-based models predicting patients’ outcomes and taking actions on such basis, the risk of misdiagnosis must be considered and mitigated. Wearable devices can be inappropriately calibrated or malfunction, resulting in wrong readouts with a potentially significant impact on individuals and healthcare systems, contributing to increased stress and anxiety, waste of resources, and reduced quality of health provision (4). To minimize the risks associated with the use of data originating from wearables, accreditation standards should be developed (46). In the European Union, there is still no separate regulation for wearables, so these devices can be classified and certified as medical devices in accordance with Regulation (EU) 2017/745 (52). This regulation establishes the rules for market introduction, availability, and entry into service of medical devices for human use and their accessories. Medical devices are classified according to their intended purpose and their intrinsic risks (52).

#### Principle of justice

The ethical principle of justice is intimately related to fairly distributing benefits, risks, and costs (24, 53). In this context, ethical considerations concern equitable access to AI solutions. Digital health tools might not be accessible to vulnerable communities and populations (33). Furthermore, the design and development of digital health products and services might not adequately consider the needs of these populations (33). Specifically, DBs are collected through wearables that might not be accessible to everyone, reinforcing inequalities (31). Thus, ensuring fairness and equity in access is critical since the planning/design phase of digital health tools (33).

The fact that AI-based models are trained through large datasets might exacerbate embedded systematic health and social biases (20). The underrepresentation of certain groups in training and testing datasets used to create and validate AI models is another factor contributing to bias in AI (29). Therefore, the design of AI algorithms based on PROs and DBs should consider the need for data debiasing and contextualization to avoid discrimination and stigmatization of more vulnerable groups (54).

Training AI algorithms with real-world data and high-quality scientific evidence data might reduce bias and improve clinical practices (39).

Accordingly, the Montréal Declaration (37) states that AI systems must be designed and trained to create a just and equitable society, ensuring that it does not create, reinforce, or reproduce discrimination. Furthermore, other regulatory efforts refer to the same recommendation (22, 55–57).

These concerns might be addressed from the beginning of the design process, for instance, by ensuring a multidisciplinary and participatory approach (58).

#### Principles of transparency and accountability

Respect for the principles of transparency and accountability should also be highlighted in the context of AI adoption in healthcare (25).

Transparency consists of opening the entire process of AI adoption to public scrutiny, including decision-making and developed actions (4). Accountability refers to the obligation to explain and justify the actions taken (59) and to establish mechanisms to ensure that responsibilities are traced and held (33). These two principles are especially critical when analyzing ethical, legal, and social issues regarding AI processes (51).

AI transparency is closely linked to traceability and explainability/interpretability (51). Traceability refers to transparently documenting the whole AI development process, including monitoring the model's performance in real-world practice after deployment (51). Meanwhile, explainability or interpretability refers to the capacity to comprehend and convey AI systems’ reasoning, choices, and actions. This is especially crucial in the healthcare industry, as AI-powered systems have the potential to impact critical decisions like prognosis and diagnosis (60).

Low interpretability is usually related to more complex models, such as neural networks, where comprehending the relation between the input and the output is challenging and may raise concerns (61–63). For instance, a physician may know that a patient is at risk without fully understanding the underlying factors contributing to that risk and, consequently, how to address it effectively (36).

The “black box” nature of AI-based models may reflect a lack of transparency, even to developers (39, 43), which may result in public mistrust and social rejection of AI implementation at consequent opportunity costs (4). In addition, with less interpretable models, identifying and correcting possible sources of bias can be more challenging (36, 43, 63). Therefore, transparency regarding structures, underlying models, stakeholders, and their interests is a key value (33) that contributes to trustworthiness (25) and reinforces respect for other ethical principles (64).

Notwithstanding, AI-based models’ continuous learning and adaption over time involves keeping up with new data (36), which reflects changes in populations, technology, and care processes (39). Therefore, subsequent evaluations are necessary even if an AI algorithm has been subjected to a rigorous initial evaluation (39).

The United States Food and Drug Administration (FDA) (65) presented innovative regulations to monitor modifications in adaptive models. These regulations require manufacturers to prespecify the anticipated modifications and establish protocols to address possible risks from these modifications (65). Furthermore, the involvement of the public (including patients and providers) in the design of AI-based models may help mitigate these risks (4).

Accountability is also central to trustworthy and applicable AI in the healthcare field. Nonetheless, there are still legal gaps in the current national and international laws about who should be responsible or liable for errors or malfunctions of AI systems (51). Since there are many actors engaged in the development and implementation of AI solutions for healthcare, such as patients, healthcare professionals, developers, and healthcare organizations, it is challenging to define roles and responsibilities (4, 51). Structured frameworks and mechanisms are essential for appropriately assigning responsibility to all participants involved in the healthcare-AI workflow, incentivizing the implementation of comprehensive measures and best practices to mitigate errors and reduce harm to patients (66). Another recommended approach to reinforce accountability could consist of regular audits and risk assessments to determine the level of regulatory oversight required for a specific AI tool (51). These assessments should encompass the entire AI pipeline, from data collection and development to pre-clinical stages, deployment, and ongoing use (51).

Table 1 synthesizes the key themes identified in the narrative review, mapping the specific ethical considerations within each principle to the corresponding e-Delphi items developed for the consensus-building phase.

**Table 1:** Ethical principles, themes from literature review, and corresponding e-delphi items.

Ethical principle	Themes from literature review	e-Delphi items
Principle of autonomy	Data control and informed consent	• Implement granular consent mechanisms, allowing individuals to authorize specific uses of their data.^(*)^ • Provide individuals control over their data, including the ability to view, manage, modify, and withdraw consent at any time.^(*)^ • Standardize consent processes across national and European levels to ensure uniformity and ease of patient authorization. • Adopt dynamic and continuously updated consent models, ensuring clear and tailored communication about data collection, usage, and implications. • Periodically review and reaffirm consent mechanisms to ensure sustained autonomy in long-term data collection. • Inform individuals about how their data was used in AI model development, including details on what data was included or excluded. • Use AI to support patient advocacy by empowering individuals to analyze their own data, identify trends, and generate reports for healthcare consultations.
	Effective communication tailored to health literacy levels	• Ensure consent processes are comprehensible and adaptable to different levels of health literacy, promoting equitable patient engagement. • Establish transparent protocols for patient communication when AI-driven decisions impact their health, ensuring information is clear, accurate, and precise.
	Transparency and patient rights in AI decision-making	• Grant patients the “right to explanation,” enabling them to understand the rationale behind AI-generated recommendations.^(*)^ • Share information about the algorithm's performance, the measures taken to identify and prevent errors, and the repercussions for patients’ health if the AI algorithm is biased or incorrect.^(*)^ • Establish mechanisms that allow patients to contest AI-generated insights or decisions they disagree with.^(*)^
Principle of beneficence	Individual and public benefits	• Validate AI model effectiveness across different patient groups and stakeholders at multiple checkpoints over time, rather than relying solely on initial assessments.^(*)^ • Assess AI system benefits from multiple stakeholder perspectives, recognizing distinct priorities among patients, healthcare providers, and administrators.^(*)^ • Uphold human oversight as a core principle, ensuring AI-driven healthcare decisions are reviewed by qualified professionals.
	Risk-benefit assessment	• Implement a comprehensive evaluation framework that tracks performance at each stage of the AI pipeline (data acquisition, preprocessing, modeling, and deployment) to identify sources of deviation and necessary adjustments. • Establish protocols for ongoing assessment and validation of ML models to ensure they continue to deliver beneficial outcomes. • Implement continuous model and data drift monitoring with predefined thresholds to trigger model retraining or resampling when necessary. • Regularly update and recalibrate models to reflect changes in patient populations and healthcare practices. • Support responsible AI development by implementing audits and certifications without stifling innovation. Provide mechanisms for developers to promote responsible AI development, reducing barriers without compromising safety or ethical standards.^(*)^
	Interoperability and data sharing	• Ensure interoperability between PROs and DBs platforms with other healthcare systems, incorporating real-time data processing capabilities to facilitate timely and informed decision-making for both clinicians and patients.^(*)^ • Facilitate secure and ethical patient data sharing across organizations to enhance accuracy and reliability in patient care while safeguarding privacy.
Principle of non-maleficence	Data privacy, security and compliance	• Safeguard human security through robust cybersecurity measures, preventing unauthorized data access and mitigating potential misuse. • Train staff on cybersecurity best practices, ensuring that individuals involved in the AI lifecycle understand their responsibility to protect patient data from unauthorized access and misuse.^(*)^ • Apply “security by design” principles, throughout the AI development process, rather than relying solely on policies, to ensure that security measures are inherently built into the technology.^(*)^ • Integrate privacy and data sovereignty measures into AI solutions, ensuring that patient privacy is maintained without compromising scalability or operational efficiency. • Establish clear notification protocols in case of security breaches or data leaks, ensuring patients are promptly informed. • Establish protocols to address data breaches, including audits to determine the causes and prevent recurrence, and implement more secure procedures to protect patient data. • Implement stringent safeguards to protect stored patient data from unauthorized access. • Perform data and model audits using synthetic data to identify potential vulnerabilities, including security issues or model biases, and ensure that ethical and legal standards are maintained.^(*)^ • Implement comprehensive audits and oversight systems to ensure ongoing compliance with data protection regulations (e.g., GDPR) and the AI Act, and assess the effectiveness of decisions made based on AI outputs. • Conduct regular impact assessments to evaluate the effects of AI solutions, including patient outcomes (e.g., satisfaction, health improvements), process efficiency, and potential negative consequences (e.g., patient harm, errors). • Ensure AI applications in healthcare undergo Data Protection Impact Assessments (DPIAs) as required by GDPR Article 35.^(*)^
	Safety and error prevention	• Develop alert mechanisms that flag potential errors or inconsistencies in AI outputs, allowing for timely interventions to prevent harm. • Ensure AI systems provide explainable outputs and maintain human oversight in all healthcare decisions to prevent reliance on AI as a sole decision-maker. Ethical skepticism should guide the integration of AI into decision-making processes.
Principle of Justice	Equity, inclusion, and bias mitigation	• Actively involve diverse populations, including those from varying cultural, socioeconomic, and medical backgrounds, in the design, development, and implementation of digital health tools to ensure equitable access and outcomes. • Ensure that digital health tools are accessible to all populations, particularly vulnerable and underserved communities, with particular attention to mitigating the underuse of these tools by these groups. • Monitor and analyze the demographic profile of populations using digital health tools and take proactive steps to mitigate barriers to access for vulnerable and underserved groups.^(*)^ • Enable patients to revise their inputs over time, ensuring AI models adapt without introducing bias or undue influence.^(*)^ • Ensure diverse stakeholder engagement in designing and implementing ML models to mitigate biases. • Ensure training datasets undergo rigorous bias and representativity assessments to achieve fair outcomes across all populations. • Ensure that underrepresented populations are not harmed by biases in ML models, and that measures are in place to regularly detect and correct bias during model development and deployment. • Avoid any form of positive or negative discrimination in feature selection and model design by ensuring that all categories or features used in AI models are balanced, representative, and free from bias.^(*)^
	Legal and ethical governance	• Implement legal frameworks and harmonize regulations across the EU and member states to create an environment where AI development is both ethical and just, with a focus on ensuring equal access to AI benefits.^(*)^ • Balance AI regulation to protect patients without stifling innovation, fostering an environment that supports both safety and competitiveness in the EU AI ecosystem.^(*)^ • Maintain transparency and accountability in AI systems so patients understand how their data is used and trust that it contributes to fair, unbiased outcomes.
Principles of transparency and accountability	Transparency and disclosure	• Implement measures to ensure that AI development and deployment processes, including decision-making criteria and model performance, are well-documented and openly shared with relevant stakeholders. • Regularly publish publicly accessible reports on AI system performance to build trust and demonstrate continuous improvement. • Make information about the databases and statistical methodologies used in AI training and validation publicly available. • Encourage transparent reporting of AI-related errors and unintended consequences by both healthcare providers and AI developers. • Ensure that all stakeholders, including patients and healthcare providers, are informed about any errors or malfunctions that may negatively impact patient care.^(*)^ • Promptly disclose any issues related to patient data usage, such as breaches or misuse, to maintain trust in AI applications.^(*)^
	Interpretability and user education	• Ensure AI models provide interpretable outputs, including the probability and uncertainty of results, to enhance user understanding. • Enhance the interpretability of ML models to ensure healthcare providers and patients can understand and trust their outputs. • Integrate Machine Learning Operations (MLOps) best practices in AI-driven healthcare solutions to ensure robust, efficient, and transparent model development, deployment, and maintenance.^(*)^ • Implement and regularly update training programs for users of AI healthcare tools to ensure they understand the principles of transparency and accountability. • Ensure transparency in AI integration, informing both patients and healthcare professionals of its role and impact in decision-making.
	Human oversight	• Ensure AI outputs support, rather than dictate, healthcare decisions.^(*)^ • Ensure human oversight is incorporated into AI systems to guarantee accountability and maintain a role for healthcare providers in decision-making.
	Accountability and governance	• Establish regular and well-defined audit intervals for AI systems that ensure continuous compliance with ethical and legal standards, while maintaining a balanced approach that upholds accountability without hindering innovation in AI development. • Conduct regular audits and risk assessments to identify and mitigate potential errors and malfunctions. • Establish data lineage protocols to track all data actions, ensuring a non-repudiation method for authorized actors involved in AI healthcare workflows.^(*)^ • Clearly define the responsibility of regulatory bodies and oversight entities in the continuous evaluation and governance of AI models in healthcare.

The e-Delphi items were developed based on themes identified in the literature review and subsequently presented to experts for consensus. Items highlighted with an asterisk (*) were added or modified based on expert suggestions during the first round of the e-Delphi process.

### Phase 2: modified e-Delphi study

The modified e-Delphi study included 23 experts in the first round and 27 experts in the second and final round, from diverse professional backgrounds and demographics. Table 2 summarizes the final round participants’ characteristics.

**Table 2:** Participant characteristics (final round, *n* = 27).

Category	Subgroup	*n*	%
Professional background	Professors/researchers	9	33.3%
	Technology company members	8	29.6%
	Healthcare professionals	3	11.1%
	Data protection Officers/regulators	3	11.1%
	Health administrators	2	7.4%
	Patient representative	2	7.4%
Gender	Male	17	63.0%
	Female	10	37.0%
Age group	18–30	3	11.1%
	31–40	6	22.2%
	41–50	9	33.3%
	51–60	5	18.5%
	61–70	2	7.4%
	71+	2	7.4%
Country	Portugal	21	77.8%
	Croatia	1	3.7%
	Greece	1	3.7%
	Italy	1	3.7%
	Lithuania	1	3.7%
	Spain	1	3.7%
	United Kingdom	1	3.7%

In the final round, participants rated 63 items (as shown in Table 1). Of these, 55 items (87.3%) reached a high level of consensus, defined as ≥80% agreement, with a median rating of 5 (“strongly agree”). The 8 items that did not reach consensus are the following:

Make information about the databases and statistical methodologies used in AI training and validation publicly available. (Agreement: 77.8%)
Assess AI system benefits from multiple stakeholder perspectives, recognizing distinct priorities among patients, healthcare providers, and administrators. (Agreement: 77.8%)
Enable patients to revise their inputs over time, ensuring AI models adapt without introducing bias or undue influence. (Agreement: 74.1%)
Periodically review and reaffirm consent mechanisms to ensure sustained autonomy in long-term data collection. (Agreement: 74.1%)
Inform individuals about how their data was used in AI model development, including details on what data was included or excluded. (Agreement: 74.1%)
Establish mechanisms that allow patients to contest AI-generated insights or decisions they disagree with. (Agreement: 70.4%)
Use AI to support patient advocacy by empowering individuals to analyze their own data, identify trends, and generate reports for healthcare consultations. (Agreement: 70.4%)
Regularly publish publicly accessible reports on AI system performance to build trust and demonstrate continuous improvement. (Agreement: 66.7%)

Table 3 presents the recommendations that reached a high level of consensus (≥80%) along with their median ratings and interquartile ranges (IQR). These recommendations, grouped according to core ethical principles, reflect the expert consensus on the essential aspects of integrating PROs and DBs in AI healthcare solutions. The table highlights key considerations for autonomy, beneficence, non-maleficence, justice, transparency, and accountability, providing actionable guidance for ethical and effective implementation.

**Table 3:** Key recommendations for integrating PROs and DBs in AI healthcare solutions.

Principle	Recommendation	Level of Agreement (%)	Median (IQR)
Autonomy	Ensure consent processes are comprehensible and adaptable to different levels of health literacy, promoting equitable patient engagement.	92.6%	5 (5–5)
	Establish transparent protocols for patient communication when AI-driven decisions impact their health, ensuring information is clear, accurate, and precise.	92.6%	5 (4–5)
	Adopt dynamic and continuously updated consent models, ensuring clear and tailored communication about data collection, usage, and implications.	88.9%	5 (4–5)
	Grant patients the “right to explanation,” enabling them to understand the rationale behind AI-generated recommendations.	88.9%	5 (4–5)
	Share information about the algorithm's performance, the measures taken to identify and prevent errors, and the repercussions for patients’ health if the AI algorithm is biased or incorrect.	88.9%	4 (4–5)
	Provide individuals control over their data, including the ability to view, manage, modify, and withdraw consent at any time.	85.2%	5 (4–5)
	Standardize consent processes across national and European levels to ensure uniformity and ease of patient authorization.	85.2%	5 (4–5)
	Implement granular consent mechanisms, allowing individuals to authorize specific uses of their data.	81.5%	5 (4–5)
Beneficence	Validate AI model effectiveness across different patient groups and stakeholders at multiple checkpoints over time, rather than relying solely on initial assessments.	100%	5 (4–5)
	Establish protocols for ongoing assessment and validation of ML models to ensure they continue to deliver beneficial outcomes.	100%	5 (4–5)
	Support responsible AI development by implementing audits and certifications without stifling innovation. Provide mechanisms for developers to promote responsible AI development, reducing barriers without compromising safety or ethical standards.	96.3%	5 (4–5)
	Implement a comprehensive evaluation framework that tracks performance at each stage of the AI pipeline (data acquisition, preprocessing, modeling, and deployment) to identify sources of deviation and necessary adjustments.	96.3%	5 (4–5)
	Uphold human oversight as a core principle, ensuring AI-driven healthcare decisions are reviewed by qualified professionals.	92.6%	5 (4–5)
	Ensure interoperability between PROs and DBs platforms with other healthcare systems, incorporating real-time data processing capabilities to facilitate timely and informed decision-making for both clinicians and patients.	88.9%	5 (4–5)
	Facilitate secure and ethical patient data sharing across organizations to enhance accuracy and reliability in patient care while safeguarding privacy.	88.9%	5 (4–5)
	Implement continuous model and data drift monitoring with predefined thresholds to trigger model retraining or resampling when necessary.	88.9%	4 (4–5)
	Regularly update and recalibrate models to reflect changes in patient populations and healthcare practices.	88.9%	5 (4–5)
Non-maleficence	Safeguard human security through robust cybersecurity measures, preventing unauthorized data access and mitigating potential misuse.	100%	5 (5–5)
	Train staff on cybersecurity best practices, ensuring that individuals involved in the AI lifecycle understand their responsibility to protect patient data from unauthorized access and misuse.	100%	5 (5–5)
	Establish protocols to address data breaches, including audits to determine the causes and prevent recurrence, and implement more secure procedures to protect patient data.	100%	5 (4–5)
	Develop alert mechanisms that flag potential errors or inconsistencies in AI outputs, allowing for timely interventions to prevent harm.	100%	5 (4–5)
	Conduct regular impact assessments to evaluate the effects of AI solutions, including patient outcomes (e.g., satisfaction, health improvements), process efficiency, and potential negative consequences (e.g., patient harm, errors).	100%	5 (5–5)
	Apply “security by design” principles, throughout the AI development process, rather than relying solely on policies, to ensure that security measures are inherently built into the technology.	96.3%	5 (4–5)
	Integrate privacy and data sovereignty measures into AI solutions, ensuring that patient privacy is maintained without compromising scalability or operational efficiency.	96.3%	5 (4–5)
	Implement stringent safeguards to protect stored patient data from unauthorized access.	96.3%	5 (5–5)
	Perform data and model audits using synthetic data to identify potential vulnerabilities, including security issues or model biases, and ensure that ethical and legal standards are maintained.	96.3%	5 (4–5)
	Implement comprehensive audits and oversight systems to ensure ongoing compliance with data protection regulations (e.g., GDPR) and the AI Act, and assess the effectiveness of decisions made based on AI outputs.	92.6%	5 (4–5)
	Ensure AI applications in healthcare undergo Data Protection Impact Assessments (DPIAs) as required by GDPR Article 35.	92.6%	5 (4–5)
	Ensure AI systems provide explainable outputs and maintain human oversight in all healthcare decisions to prevent reliance on AI as a sole decision-maker. Ethical skepticism should guide the integration of AI into decision-making processes.	88.9%	5 (4–5)
	Establish clear notification protocols in case of security breaches or data leaks, ensuring patients are promptly informed.	81.5%	5 (4–5)
Justice	Maintain transparency and accountability in AI systems so patients understand how their data is used and trust that it contributes to fair, unbiased outcomes.	96.3%	5 (4–5)
	Actively involve diverse populations, including those from varying cultural, socioeconomic, and medical backgrounds, in the design, development, and implementation of digital health tools to ensure equitable access and outcomes.	92.6%	5 (4–5)
	Ensure that digital health tools are accessible to all populations, particularly vulnerable and underserved communities, with particular attention to mitigating the underuse of these tools by these groups.	92.6%	5 (4–5)
	Ensure training datasets undergo rigorous bias and representativity assessments to achieve fair outcomes across all populations.	92.6%	5 (4–5)
	Ensure that underrepresented populations are not harmed by biases in ML models, and that measures are in place to regularly detect and correct bias during model development and deployment.	92.6%	5 (4–5)
	Avoid any form of positive or negative discrimination in feature selection and model design by ensuring that all categories or features used in AI models are balanced, representative, and free from bias.	92.6%	5 (4–5)
	Implement legal frameworks and harmonize regulations across the EU and member states to create an environment where AI development is both ethical and just, with a focus on ensuring equal access to AI benefits.	92.6%	5 (4–5)
	Balance AI regulation to protect patients without stifling innovation, fostering an environment that supports both safety and competitiveness in the EU AI ecosystem.	92.6%	5 (4–5)
	Ensure diverse stakeholder engagement in designing and implementing ML models to mitigate biases.	88.9%	4 (4–5)
	Monitor and analyze the demographic profile of populations using digital health tools and take proactive steps to mitigate barriers to access for vulnerable and underserved groups.	81.5%	5 (4–5)
Transparency and accountability	Ensure human oversight is incorporated into AI systems to guarantee accountability and maintain a role for healthcare providers in decision-making.	100%	5 (4–5)
	Establish regular and well-defined audit intervals for AI systems that ensure continuous compliance with ethical and legal standards, while maintaining a balanced approach that upholds accountability without hindering innovation in AI development.	96.3%	4 (4–5)
	Conduct regular audits and risk assessments to identify and mitigate potential errors and malfunctions.	96.3%	4 (4–5)
	Implement and regularly update training programs for users of AI healthcare tools to ensure they understand the principles of transparency and accountability.	96.3%	5 (4–5)
	Ensure transparency in AI integration, informing both patients and healthcare professionals of its role and impact in decision-making.	96.3%	5 (4–5)
	Implement measures to ensure that AI development and deployment processes, including decision-making criteria and model performance, are well-documented and openly shared with relevant stakeholders.	92.6%	5 (4–5)
	Encourage transparent reporting of AI-related errors and unintended consequences by both healthcare providers and AI developers, fostering a culture of transparency and accountability.	92.6%	4 (4–5)
	Integrate MLOps best practices in AI-driven healthcare solutions to ensure robust, efficient, and transparent model development, deployment, and maintenance.	92.6%	4 (4–5)
	Ensure AI outputs support, rather than dictate, healthcare decisions.	92.6%	5 (4–5)
	Clearly define the responsibility of regulatory bodies and oversight entities in the continuous evaluation and governance of AI models in healthcare.	92.6%	5 (4–5)
	Ensure that all stakeholders, including patients and healthcare providers, are informed about any errors or malfunctions that may negatively impact patient care.	88.9%	5 (4–5)
	Ensure AI models provide interpretable outputs, including the probability and uncertainty of results, to enhance user understanding.	88.9%	5 (4–5)
	Enhance the interpretability of ML models to ensure healthcare providers and patients can understand and trust their outputs.	88.9%	5 (4–5)
	Establish data lineage protocols to track all data actions, ensuring a non-repudiation method for authorized actors involved in AI healthcare workflows.	88.9%	4 (4–5)
	Promptly disclose any issues related to patient data usage, such as breaches or misuse, to maintain trust in AI applications.	85.2%	5 (4–5)

**Figure 2:**
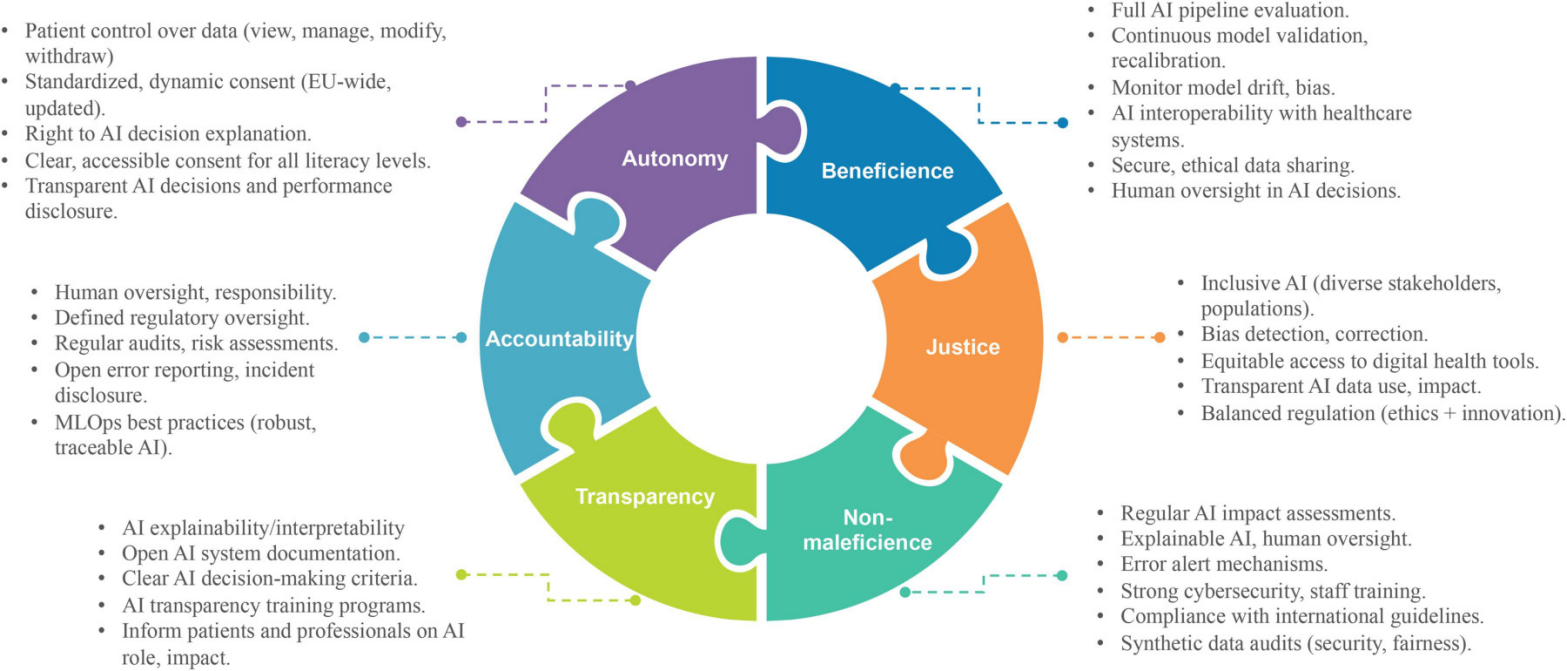
Experts’ recommendations for integration of PROs and DBs in AI-based models and their respective correspondence to the main ethical principles as resulting from our narrative literature review [author's elaboration].

Figure 2 provides a visual summary of the main experts’ recommendations for the integration of PROs and DBs in AI-based models and their respective correspondence to the main ethical principles as resulting from our narrative literature review.

## Discussion

This study presents the first European expert consensus specifically addressing the ethical integration of PROs and DBs in AI healthcare models, exploring their ethical, legal, and social considerations. While existing literature extensively covers general AI ethics principles, these findings reveal unique considerations that arise specifically from patient-generated health data. The 55 consensus recommendations provide targeted guidance for the distinct challenges posed by continuous patient data collection.

The findings highlight a strong consensus among participants regarding the importance of upholding key ethical principles in this context. The healthcare landscape is indeed undergoing a significant transformation with the increasing integration of AI across various domains, further fueled by the growing availability and utilization of PROs and DBs. PROs capture patients’ perspectives on their health status, symptoms, and functional abilities, offering valuable subjective insights, while DBs, from wearable sensors and other technologies, provides continuous and objective data. The convergence of AI with these rich data sources holds immense potential to revolutionize healthcare by enabling personalized interventions, predictive analytics, and more efficient clinical workflows. However, this powerful integration also brings forth a complex array of ethical, legal, and social implications (ELSIs) that demand careful consideration to ensure responsible innovation and safeguard patient rights (67).

### Principle of autonomy

Regarding the principle of autonomy, participants emphasized the need for individuals to maintain control over their data, including the ability to view, manage, modify, and withdraw consent at any time. This aligns with the ethical principle of respecting and supporting autonomous decisions (24). The importance of clear and tailored communication, along with ensuring understandable consent processes that accommodate varying levels of health literacy, was strongly affirmed. This is consistent with the notion that AI adoption should contribute to patient-centered healthcare, where patients are involved in decision-making processes and are well-informed about the purpose, risks, benefits, and alternatives of any intervention. Research consistently highlights informed consent and patient autonomy as paramount ethical considerations in this rapidly evolving field (23). Patients must be informed about the role of AI in their care by healthcare professionals and technology developers and should have the freedom to decline its involvement if they feel uncomfortable. This perspective aligns with the understanding that AI should serve as a tool to augment, rather than replace, the essential human interaction and empathy that are integral to quality patient care (68, 69).

Several recommendations focused on enhancing the informed consent process. The call for standardized consent across national and European levels reflects the need for uniformity and ease of patient authorization. Furthermore, the emphasis on dynamic and continuously updated consent models, along with transparent protocols for patient communication, underscores the importance of ensuring continued comprehension and voluntariness. The recognition that informed consent comprehension can be influenced by factors such as age, gender, health literacy, and contextual factors (34) highlights the need for adaptable consent processes. This is particularly crucial for collecting DBs, where continuous passive data collection can potentially limit autonomy due to unanticipated data treatment.

The concept of a “right to explanation,” enabling patients to understand the reasoning behind AI system recommendations, received substantial support (88.9%). This aligns with the broader principle of transparency, which is crucial for building trust in AI-based healthcare models (63). Sharing information about the algorithm's performance, error prevention measures, and potential repercussions for patients’ health in cases of bias or errors was also considered important. This “right to explanation” has gained significant traction, emphasizing the need for explainable AI (XAI) to foster patient trust and informed decisions (67, 70).

### Principle of beneficence

The principle of beneficence, focused on promoting well-being and balancing benefits against risks and costs, also garnered strong agreement. Participants strongly supported the implementation of a comprehensive evaluation framework to track AI pipeline performance (96.3%) and the establishment of protocols for ongoing assessment and validation of machine learning models (100%). This reflects the understanding that the continuous learning characteristic of AI algorithms necessitates continuous evaluation to ensure ongoing patient benefit (71).

Recommendations emphasized the importance of validating AI model effectiveness across diverse patient groups and stakeholders over time rather than relying solely on initial assessments (100%). The need for continuous model and data drift monitoring, along with regular updates and recalibration of models, was also highlighted (88.9%). Ensuring interoperability between PROs and DBs platforms with other healthcare systems and promoting secure and ethical data sharing across organizations were identified as key to improving the accuracy and reliability of patient care (88.9%). These measures align with the potential benefits of integrating PROs and DBs in AI-based models, such as providing personalized care, anticipating healthcare needs (individual benefit), cost reduction, and increased quality performance (public benefit). Robust frameworks for evaluating AI pipeline performance are essential to ensure beneficence (72). Continuous assessment is crucial due to AI's learning nature (73).

The recommendation for human oversight was strongly endorsed (92.6%), reinforcing the importance of qualified professionals reviewing AI-driven decisions in healthcare. Furthermore, participants acknowledged the need to balance audits and certifications with support mechanisms for developers to promote responsible AI development without stifling innovation (96.3%). While AI offers significant benefits, human oversight remains critical to ensure patient safety and maintain the human aspects of care, like empathy (23). Patients may find it difficult to accept fully automated “machine-human” medical relationships (23).

### Principle of non-maleficence

The principle of non-maleficence, centered on avoiding harm, was addressed through recommendations focused on data privacy and security, monitoring burden, and safety. Participants strongly agreed on the need to conduct regular impact assessments to evaluate the effects of AI solutions (100%) and to design AI systems that ensure explainability of their outputs (88.9%). The importance of human oversight in all healthcare decisions, with AI serving as a support tool rather than the primary determinant in healthcare decisions, was emphasized (92.6%). This aligns with the recognition that AI-based solutions may be linked to malfunctions and errors that raise questions about patient safety (74).

To mitigate potential harm, participants recommended developing alert mechanisms to flag errors or inconsistencies in AI outputs (100%) and establishing protocols to address data breaches, including audits and more secure procedures to protect patient data (100%). The need to safeguard human security alongside technological measures, prevent unauthorized access to data, and invest in comprehensive cybersecurity practices was strongly affirmed (100%). Training staff on cybersecurity best practices and implementing comprehensive audits and oversight systems to ensure ongoing compliance with data protection regulations were also deemed crucial (100%, 92.6%). Robust security protocols and cybersecurity measures are essential to prevent data breaches (75).

The principles of “security by design” and “privacy and data sovereignty” should be integrated into AI solutions (96.3%). Furthermore, AI applications in healthcare should be subject to Data Protection Impact Assessments (DPIAs) as per Article 35 of the GDPR (92.6%). The security of stored patient data must be explicitly addressed with stringent safeguards (96.3%). Data and model audits using synthetic data were recommended to identify potential vulnerabilities and ensure adherence to ethical and legal standards (96.3%).

Federated learning emerges as a promising approach that supports both beneficence and non-maleficence principles. Allowing AI models to be trained across multiple decentralized sites without exchanging the raw data itself, federated learning enables collaborative model development while preserving data privacy (76). Participants recognized that secure and ethical patient data sharing should be facilitated across organizations to enhance accuracy and reliability in patient care while safeguarding privacy (88.9%). This technique particularly benefits healthcare organizations seeking to develop robust AI models by leveraging diverse patient populations across institutions while maintaining strict data governance and sovereignty (77), which was reinforced by the recommendation to integrate privacy and data sovereignty measures into AI solutions, ensuring that patient privacy is maintained without compromising scalability or operational efficiency (96.3%).

These recommendations reflect the sensitive nature of healthcare data and the ethical concerns regarding its collection, storage, access, and sharing (67). The lack of transparency in some AI systems (“black box” phenomenon) also poses a risk of harm (78). Explainability is crucial for identifying errors and ensuring correct decisions (78–80).

### Principle of justice

The principle of justice, focused on the fair distribution of benefits, risks, and costs, was addressed through recommendations aimed at ensuring equitable access to AI solutions and mitigating biases. Participants emphasized the importance of diverse stakeholder engagement in designing and implementing machine learning models (88.9%) and actively involving diverse populations in the design, development, and implementation of digital health tools (92.6%). This aligns with the need to consider the needs of vulnerable communities and populations and to ensure fairness and equity from the planning/design phase (81).

Training datasets should undergo rigorous bias and representativity assessments (92.6%), and measures should be in place to regularly detect and correct bias during model development and deployment (92.6%). This is crucial to avoid discrimination and stigmatization of vulnerable groups, as AI-based models trained on large datasets may exacerbate embedded systematic health and social biases. Ensuring that digital health tools are accessible to all populations, particularly vulnerable and underserved communities, was also considered essential (92.6%). Identifying and mitigating biases in AI algorithms and training datasets is vital for justice and equity (82).

Transparency and accountability in AI systems were deemed necessary to allow patients to understand how their data is used and to ensure fair and unbiased outcomes (96.3%). Participants also recommended avoiding any form of positive or negative discrimination in feature selection and model design (92.6%) and implementing legal frameworks and harmonizing regulations across the EU and member states to create an environment where AI development is both ethical and just (92.6%). Balancing the enforcement of EU regulations with the competitiveness and ease of use for AI industry stakeholders in Europe was identified as a key challenge (92.6%). Diverse stakeholder engagement is crucial for achieving fair outcomes (70).

### Principles of transparency and accountability

The principles of transparency and accountability were consistently highlighted across recommendations. There was strong consensus on the need for interpretable AI outputs (88.9%), human oversight in AI systems (88.9%–100%), and well-documented development processes (92.6%). Experts agreed on the importance of implementing training programs for AI users (96.3%), and clearly defining regulatory responsibilities (92.6%).

These recommendations strongly align with established frameworks in responsible AI governance. The emphasis on interpretability addresses the “black box problem” in healthcare AI, where low interpretability raises ethical concerns around trust and clinical adoption (83).

Also, the high consensus around error reporting (92.6%) and stakeholder communication about any errors or malfunctions that may negatively impact patient care (88.9%) aligns with work by Amann et al. (80), emphasizing that explainability is not merely a technical challenge but a multidisciplinary requirement involving clinical, technical, legal, and patient perspectives.

The recommendations for regular audits and transparent documentation align with the need for traceability, explainability/interpretability, and mechanisms to ensure that responsibilities are traced and held in the development and implementation of AI solutions for healthcare (84).

Participants reached strong consensus (92.6%) on the recommendation to integrate MLOps best practices in AI-driven healthcare solutions to ensure robust, efficient, and transparent model development, deployment, and maintenance. While this recommendation is applicable across diverse healthcare AI contexts, its implementation for PROs and DBs requires addressing specific operational challenges. Patient-generated sources involve continuous or frequent data streams with inherent variability, such as when patients skip assessments, sensors malfunction, adherence fluctuates, and reporting behavior changes over time. MLOps implementation must therefore include automated pipelines for handling irregular data patterns, continuous monitoring for temporal drift in patient engagement and data quality, transparent model versioning as algorithms adapt, and explainability mechanisms that function despite heterogeneous inputs (85, 86). These practices operationalize transparency and accountability within the constraints of continuous patient data collection.

In addition to the principles discussed below, our findings implicitly address the need for adherence to FAIR data principles (Findability, Accessibility, Interoperability, and Reusability) (87).

Findability is supported through recommendations for establishing data lineage protocols (88.9%) and documenting AI development processes (92.6%). For PROs and DBs, data lineage is particularly critical given the diverse sources of data generation, from patient self-reporting through various digital interfaces to continuous streams from wearable devices and sensors. These practices ensure that data can be discovered and identified through proper metadata and persistent identifiers, which are critical for tracing data provenance in AI systems (88).

Accessibility is reflected in recommendations for transparent data sharing across organizations (88.9%) and ensuring that data access is secured through appropriate authentication and authorization protocols. Given the highly personal nature of PRO data and the continuous, granular health information captured by DBs (89), the emphasis on protocols to address data breaches (100%) and implementation of comprehensive audits (92.6%) is particularly relevant. These measures support controlled accessibility that balances the need for multi-site collaboration with the heightened privacy requirements inherent to patient-generated health data (90).

Interoperability received explicit support (88.9%) through recommendations to ensure PROs and DBs platforms can communicate with other healthcare systems. This facilitates the exchange and integration of data across different platforms and applications, enhancing the potential for comprehensive analysis while maintaining semantic integrity (91).

Reusability is embedded in recommendations for comprehensive documentation of AI development processes (92.6%) and establishing protocols for ongoing assessment and validation (100%).

The integration of FAIR principles with ethical considerations creates a robust framework for responsible AI development in healthcare, supporting both innovation and patient protection (82).

The 55 recommendations represent a comprehensive ethical ideal. However, resource-constrained settings may need to prioritize implementation. We suggest that items achieving 100% consensus (*n* = 8 items), focused on cybersecurity, data breaches, impact assessments, and human oversight, represent non-negotiable minimum requirements. Items with 92%–96% consensus represent high-priority aspirational standards. Organizations should assess their readiness and prioritize implementation accordingly, while working toward comprehensive adoption over time.

Also, effective implementation requires translating these principles into actionable steps for different stakeholders. Future research should evaluate implementation of these recommendations through pilot studies, assess their impact on patient outcomes and system performance, and identify barriers to adoption across different healthcare settings and regulatory contexts.

Beyond the core ethical principles analyzed in our study, there are complementary considerations that merit attention in future research on integrating PROs and DBs in AI healthcare solutions. One such consideration relates to the data collection burden itself.

The potential burden on individuals responding to PRO questionnaires (92), represents an important non-maleficence consideration that deserves further investigation. Factors such as the length or formatting of the questionnaire, the literacy level of respondents, and the mode of administration are potential factors related to response burden (93). Similarly, for DBs, there may be associated burdens related to carrying or safeguarding electronic devices and the discomfort some patients experience from being tracked (94).

Additionally, tight follow-up protocols and subsequent interventions may influence patient responses, potentially compromising data validity and the benefits of AI algorithms. For instance, individuals may deliberately omit a response to avoid contact or specific clinical interventions (36). These considerations highlight the critical importance of involving healthcare professionals in designing tailored interventions that respect individual patient preferences and behaviors, ensuring inclusive and ethical implementation (95).

These dimensions of data collection burden provide relevant context to our findings on ethical integration of PROs and DBs in AI healthcare solutions and could be further elaborated in future research.

### Areas of expert divergence

While the high consensus rate (87.3% of items reaching ≥80% agreement) demonstrates strong alignment among experts, the eight items that did not reach the inclusion threshold merit analytical attention. These non-consensus items can be grouped thematically into three clusters: (a) transparency and public disclosure, (b) patient agency and contestation, and (c) consent sustainability and data governance.

Regarding transparency and public disclosure, the items on making AI training databases and statistical methodologies publicly available (77.8%) and regularly publishing publicly accessible performance reports (66.7%) suggest that while experts value transparency as a principle, there may be concerns about the feasibility, intellectual property implications, or potential misinterpretation of technical information when made broadly available (96, 97).

In the domain of patient agency, the items on enabling patients to contest AI-generated insights (70.4%) and using AI to support patient advocacy through self-data analysis (70.4%) represent future-facing recommendations that may not yet have established implementation pathways. Expert caution regarding these items may reflect concerns about patient safety when engaging directly with complex AI outputs, or about the readiness of current AI systems to support such interactions safely and meaningfully (98). These items nonetheless represent important aspirational directions for future research and system design.

A particularly instructive comparison emerges between two consent-related items. The included recommendation to “adopt dynamic and continuously updated consent models” (88.9%) focuses on proactively embedding flexibility into the design of consent processes, whereas the excluded item on “periodically reviewing and reaffirming consent mechanisms” (74.1%) implies ongoing institutional audit obligations. The lower agreement on the latter may indicate that some experts viewed periodic institutional review as operationally burdensome or potentially redundant given existing regulatory requirements, such as GDPR's data protection impact assessments. This distinction suggests a preference among experts for building consent adaptability into system architecture rather than relying on periodic external review mechanisms (99).

Similarly, the items on enabling patients to revise their inputs over time (74.1%) and assessing AI system benefits from multiple stakeholder perspectives (77.8%) approached the consensus threshold, suggesting that these are areas of emerging but not yet consolidated agreement. The item on informing individuals about how their data was used in AI model development (74.1%) also highlights the ongoing tension between comprehensive transparency and practical feasibility in complex data processing pipelines (100, 101). These non-consensus items collectively identify important boundaries of current expert agreement and represent priority areas for future Delphi studies as the field matures and implementation experience accumulates.

### Strengths and limitations

Although several studies address the ethical considerations in the use of artificial intelligence in healthcare (20, 102–106), this is, to the best of our knowledge, the first study to comprehensively map and to achieve European expert consensus specifically on PROs and DBs integration in AI healthcare models. This targeted approach distinguishes our work from broader AI ethics frameworks and provides actionable guidance for the specific challenges that developers, researchers, and policymakers face when integrating PROs and DBs in AI-based healthcare models. This novel contribution enhances the relevance of the findings beyond this specific context. While the analysis is centered on PROs and DBs, the identified considerations are relevant to a broad range of health-related data processing scenarios. As such, they can serve as a foundational ethical and operational framework for policymakers, developers, researchers, and practitioners working across different health technologies.

However, the study is subject to limitations regarding the composition of the expert panel. First, the panel reflects significant geographic concentration, with 77.8% of participants (21 of 27) from Portugal and all participants from European countries (Portugal, Spain, Italy, Greece, Croatia, Lithuania, and the United Kingdom). This concentration has important implications for the generalizability of findings. While many ethical principles are universal, their interpretation and implementation are shaped by regional regulatory frameworks (particularly GDPR and the EU AI Act in this study), healthcare system structures, cultural norms regarding privacy and autonomy, and resource availability (107). Consequently, healthcare systems in other regions may interpret or prioritize these ethical considerations differently. Future work should explicitly seek geographic diversity, particularly in low- and middle-income countries (LMICs), to test whether these consensus recommendations hold across different regulatory, cultural, and healthcare contexts, or whether region-specific adaptations are necessary.

Second, regarding the expertise within the panel, it is important to note that while invitations were extended to external legal scholars and ethicists, these experts were unable to participate. Consequently, no formally trained ethicists or legal scholars were included as distinct professional categories within the external voting panel, although several participants possessed bioethics knowledge and professional roles as data protection officers/regulators. However, to mitigate this limitation, the study design, framework development, and interpretation of findings were closely supervised by the authorship team, which includes a Professor of Health Law and expert in health ethics. While this internal expertise ensured robust normative oversight, the inclusion of a dedicated external legal and ethical panel in future iterations would further deepen the jurisprudential analysis of these recommendations.

Finally, while the study included two patient representatives, a more comprehensive understanding would benefit from larger and more diverse patient representation across different health conditions, demographic backgrounds, and geographic contexts, as well as direct involvement of the wider public, who may have different perspectives on data privacy, AI trust, and acceptable trade-offs between innovation and protection compared to professional experts and organized patient advocates. Including these perspectives more comprehensively in future research will be essential for ensuring that ethical frameworks reflect the priorities, concerns, and values of those most directly affected by AI-driven healthcare (108).

Methodologically, the use of a e-Delphi survey provided a structured way to build expert consensus on complex normative issues (109). The process benefited from the diversity of participants across disciplines, which enriched the discussion and supported a pluralistic view of the challenges and priorities involved. Importantly, the survey was designed to ensure full anonymity; no login credentials or personal information that could allow the identification of the participants were collected at any point. While this approach safeguarded privacy and encouraged open contributions, it also introduced certain limitations.

Specifically, the research team could not track individual participation across rounds, limiting the ability to assess response consistency, drop-out rates, or potential shifts in opinion. Furthermore, this design precluded the analysis of whether disagreement patterns on non-consensus items varied systematically by country of origin or professional background. Future studies employing pseudonymized or trackable participation may enable such subgroup analyses, providing further insight into the sources of divergence. However, the high consensus levels achieved (87.3% of items reaching ≥80% agreement), the consistency of response patterns across ethical domains suggest that this limitation did not substantially undermine the validity of consensus findings.

## Conclusion

The large-scale processing of patient-generated health data, particularly PROs and DBs, by ML algorithms presents unique ethical challenges. This study provides the first European consensus framework specifically designed for these data types, analyzing ethical considerations through traditional bioethical principles complemented by transparency and accountability.

In this study, we have analyzed the ethical, legal, and social considerations regarding integrating PROs and DBs in ML models based on the four traditional bioethical principles of respect for autonomy, beneficence, non-maleficence, and justice, complemented by the principles of transparency and accountability.

Despite our analysis being focused on PROs and DBs, the identified considerations can be used as a baseline for a wide range of contexts of health-related data processing, supporting policymakers, developers, researchers, and practitioners in tackling these potential risks.

It is crucial to consider the ethical threats and risks associated with integrating sensitive personal information into ML models, explore its potential impact on every aspect of people's lives, and build regulation systems that enhance and protect their rights.

This study offers a timely and relevant contribution to ongoing efforts to align technological innovation with ethical and societal values. It provides a structured, consensus-driven foundation that can inform both policy development and practical implementation of AI in health systems that increasingly rely on diverse and dynamic data sources.

## Data Availability

The original contributions presented in the study are included in the article/Supplementary Material, further inquiries can be directed to the corresponding author.
